# 

**Published:** 1863-06

**Authors:** 


### BOOKS RECEIVED.

*Transactions of the Medical Association of Southern Central New York, at the* 12*th*, 13*th*, 14*th and* 15*th Annual Meetings, held in* 1858–59–60 *and* 1861.

*Sanitary Commission No.* 65—*Department of Special Inspection of the General Hospitals of the Army—Second Report to the Committee, by* HENRY G. CLARK, M. D., *Inspector-in-Chief.*

